# Changes in the microsomal proteome of tomato fruit during ripening

**DOI:** 10.1038/s41598-019-50575-5

**Published:** 2019-10-04

**Authors:** Daniela Pontiggia, Francesco Spinelli, Claudia Fabbri, Valerio Licursi, Rodolfo Negri, Giulia De Lorenzo, Benedetta Mattei

**Affiliations:** 1 Department of Biology and Biotechnology “C. Darwin”, Sapienza University of Rome, Rome, Italy; 2 Foundation Cenci Bolognetti-Institut Pasteur, Rome, Italy; 3 Institute for Systems Analysis and Computer Science “Antonio Ruberti”, National Research Council, Rome, Italy; 4 Department of Life, Health and Environmental Sciences, University of L’Aquila, L’Aquila, Italy

**Keywords:** Plant development, Fruiting

## Abstract

The variations in the membrane proteome of tomato fruit pericarp during ripening have been investigated by mass spectrometry-based label-free proteomics. Mature green (MG30) and red ripe (R45) stages were chosen because they are pivotal in the ripening process: MG30 corresponds to the end of cellular expansion, when fruit growth has stopped and fruit starts ripening, whereas R45 corresponds to the mature fruit. Protein patterns were markedly different: among the 1315 proteins identified with at least two unique peptides, 145 significantly varied in abundance in the process of fruit ripening. The subcellular and biochemical fractionation resulted in GO term enrichment for organelle proteins in our dataset, and allowed the detection of low-abundance proteins that were not detected in previous proteomic studies on tomato fruits. Functional annotation showed that the largest proportion of identified proteins were involved in cell wall metabolism, vesicle-mediated transport, hormone biosynthesis, secondary metabolism, lipid metabolism, protein synthesis and degradation, carbohydrate metabolic processes, signalling and response to stress.

## Introduction

Tomato (*Solanum lycopersicum L*.) is a crop of high economic and nutritional value produced worldwide and the most widely used model to study different aspects of development and ripening of fleshy fruits^1^.

Fruit ripening coincides with seed maturation in the final phase of fruit development and is a highly coordinated, genetically programmed, irreversible phenomenon involving a series of physiological, biochemical, and organoleptic changes that lead to changes in colour, texture, flavour, aroma, and nutritional status^2^. It is coordinated by drastic hormonal changes and, although ethylene is considered the major hormonal regulator in climacteric fruit ripening, other hormones such as auxin and abscisic acid (ABA) take part in this process^3^. During fruit development, the concentration of carbohydrates, amino acids, and organic acids diminishes immediately after fruit setting and is partially recovered during fruit ripening^4^ with increased sugar levels, starch hydrolysis, decreased acidity^1^, pulp softening^5^, aromatic component and color development^6^.

The tomato genome has been recently sequenced and annotated^7^ and a number of post-genomic approaches have been used to gain insights into molecular networks controlling fruit development and ripening^8^. High-throughput proteomics analyses were reported recently^9–13^. Moreover, analyses of fruit transcriptomes^14,15^ and metabolome^16,17^ as well as multilevel studies integrating transcriptomics and metabolomics^4,18,19^, transcriptomics and enzyme profiles^20^, or transcriptomics, proteomics, and metabolomics^8,21^ have been performed. These studies have provided an enormous amount of data that expands our knowledge of the molecular events associated with ripening.

While many enzymes are soluble proteins, membrane-spanning or membrane-associated proteins are more challenging to proteomic analysis^22^. In this work, we obtained a proteomic profiling of the microsomal fraction of tomato pericarp at the first and last stage of the ripening process, namely mature green (MG30) and red ripe (R45)^14^, respectively, in order to elucidate the most relevant biochemical pathways associated, on the one hand, with the onset of ripening process, and, on the other hand, with the final ripe stage, with a special interest on the pathways related to cell wall metabolism, hormone biosynthesis and the production of aromatic and nutritionally important compounds. With this approach, we reliably identified 1315 proteins, among which 145 significantly varied in their abundance in the two stages of fruit ripening. The most significant variations in abundance of microsomal proteins between MG30 and R45 fruits are indeed mainly related to cell wall remodelling, vesicle trafficking, ethylene biosynthesis and lipid metabolism, as well as to glycolysis, gluconeogenesis and TCA cycle.

## Results

### Tomato membrane protein enrichment and identification

Total microsomes were prepared from the pericarp of tomato fruits sampled at 30 and 45 days post-anthesis (DPA) and corresponding to the MG30 and R45 ripening stages, respectively. These stages correspond to the first stage of ripening (MG30) and to the mature fruit (R45), respectively^14^.

**Figure 1:**
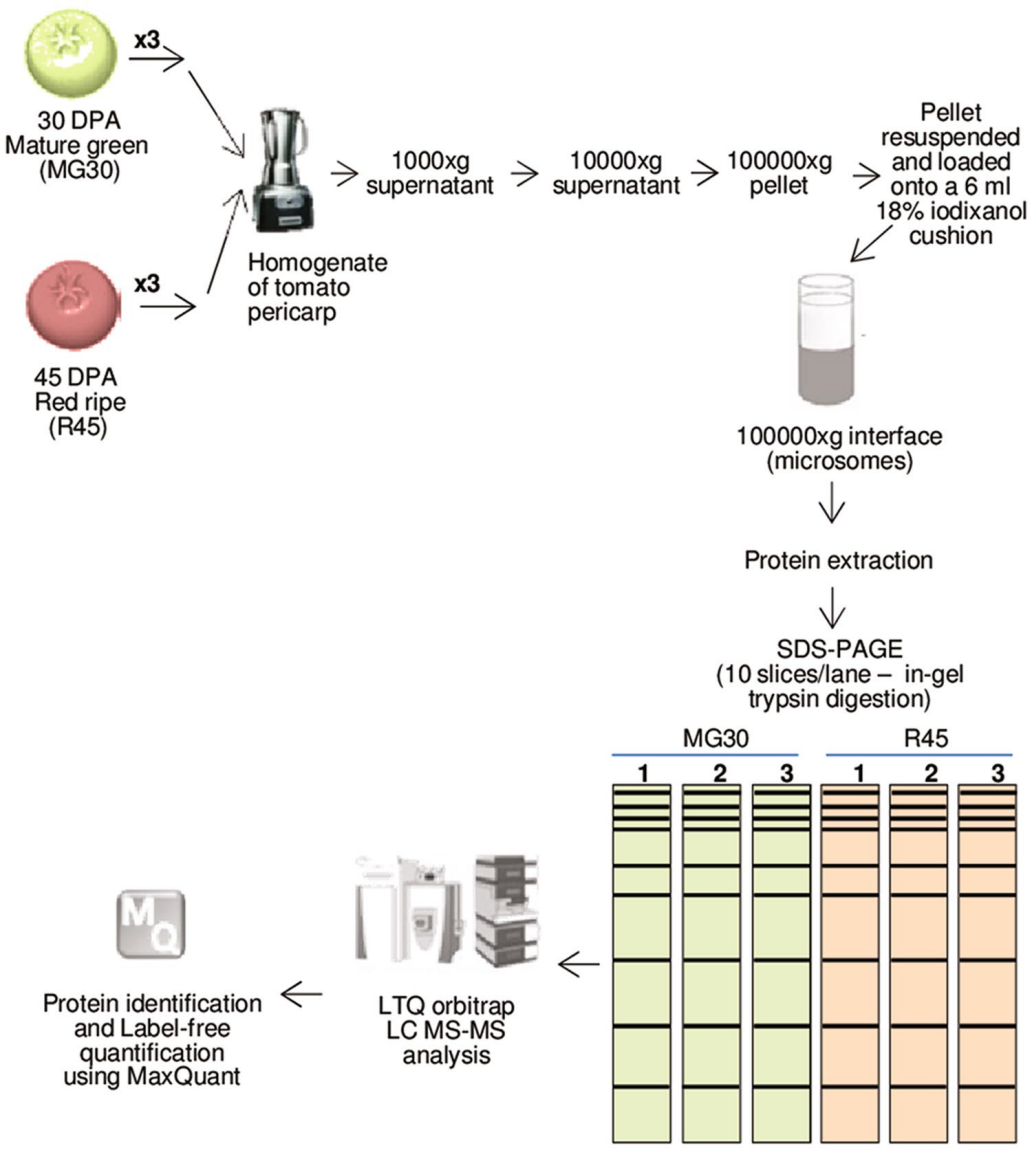
Experimental design. The experiment was performed using three independent biological replicates for each fruit stage. DPA, days post-anthesis.

**Figure 2:**
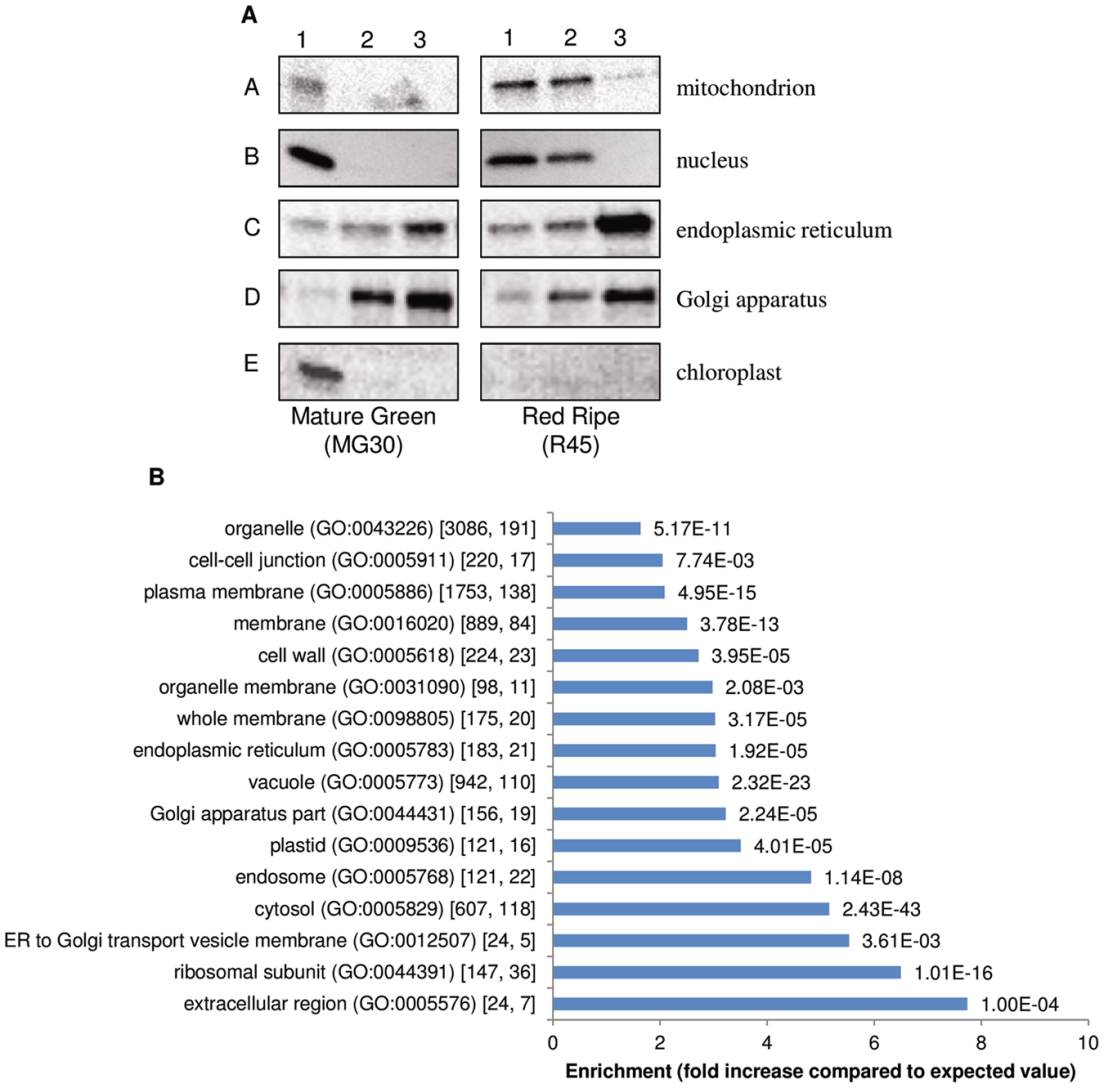
Assessment of the progressive enrichment of total tomato membranes. (**A**) Assessment of the progressive enrinchment by Western blot analysis of protein extracts. Samples (2 µg) from each stage of the purification process (1: homogenate, 2: 1000 × g supernatant, 3: total membrane) were analyzed by immunoblotting with antibodies specific for membrane markers of principal organelles (A: AOX, mitochondrion, B: H3, nucleus, C: RGP1, Golgi apparatus, D: BiP, endoplasmic reticulum, E: LHCII, chloroplast). Results are shown for one representative experiment of three performed. (**B**) Cellular component GO-term enrichment analysis for all the identified proteins (listed in Supplementary Table S1) using PHANTER analysis tool. Selected enriched GO terms are presented in this figure, and a complete list of enriched GO terms is available in Supplemental Table S2. Horizontal bars show the value of enrichment, defined as the fold increase when compared to the expected value measured on the tomato proteome, and the corresponding p-values are given next to the bars. The numbers of each GO term occurrence in the tomato proteome and in our analysis, respectively, are given inside the square brackets.

The protocol included sequential centrifugation steps (Fig. 1), using a low ionic strength buffer to maintain the organization of membrane-associated supramolecular complexes (e.g. metabolons) and better evaluate changes in membrane-associated processes during development. Western blot analysis was performed to determine the enrichment of membrane proteins in total microsomal fraction. While the initial homogenate showed the presence of the nuclear, chloroplast and mitochondrial markers, the presence of these markers was negligible in both MG30 and R45 microsomal-enriched samples; Golgi and ER markers showed instead enrichment (Fig. 2A).

To reduce sample complexity, MG30 and R45 protein fractions were subjected to monodimensional SDS-PAGE and each lane was cut into 10 slices, in-gel digested and analyzed by nano-LC-MS/MS (Fig. 1). Protein identification and quantification were performed with the MaxQuant software package^23^ against the ITAG v 2.3 protein database. In total, 1926 different proteins/protein groups (hereon indicated as proteins) were identified with a FDR < 0.01 and a minimum of one unique peptide (DataSheet S1). Stringent criteria (at least two “razor + unique” peptides, with at least one unique) were applied to obtain a high quality dataset of 1315 proteins for quantitative proteomic analysis (Table S1).

### Functional classification and expression profile analysis

The 1315 protein sequences (Table S1) were annotated according to the ITAG official annotation for the tomato genome. Among identified proteins there were the high confidence ER marker BiP (Solyc08g082820) and Golgi marker Coatomer subunit gamma (F-COP-IΥ; Solyc01g109540), proteins with a putative transmembrane region (247 proteins), of which 105 carry also a signal peptide for translocation into the endoplasmic reticulum (ER). In addition, 91 proteins carried the signal peptide only. Identified proteins were classified using the PANTHER GO classification system and GO term enrichment compared to the total tomato fruit proteome was determined (Table S2). We found particularly enriched the GO terms extracellular region, ribosomal subunits, ER to Golgi transport vesicle membranes, endosome as well as cytosol (Fig. 2B).

### Differentially expressed proteins

Quantification analysis of the 1315 identified proteins (Table S1), was performed using the label-free algorithm in MaxQuant. A total of 145 proteins (after filtering as described in the Materials and Methods) showed significantly increased or decreased abundance (>1.5-fold change); among these, the number of stage-specific proteins was 59. Proteins with fold-change <1.5 were considered invariant (159 proteins). The complete list is given in Table S3 and comprises 304 tomato unigenes. The search for counterparts in the Arabidopsis genome led to 291 Arabidopsis unigenes, since some tomato unigenes corresponded to the same Arabidopsis homolog. In addition, 425 quantified proteins did not fulfil the FDR < 0.05 criterium, and are indicated here as putatively invariant (Table S3).

**Figure 3:**
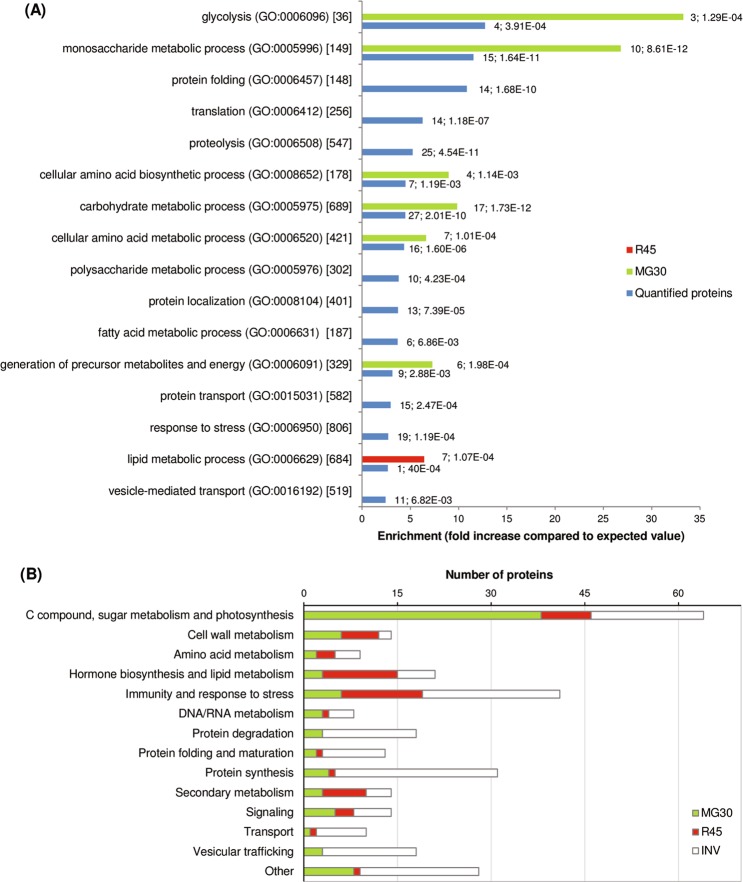
Functional classification of the tomato proteins quantified during fruit development. (**A**) Gene ontology (GO) term enrichment analysis on the quantified proteins (listed in Supplementary Table S3) using the GO annotations of biological processes in PHANTER. The x-axis represents the number of proteins in the corresponding GO class. Selected enriched GO terms are included in this figure, and a complete list of enriched GO terms is available in Supplementary Table S2. (**B**) Distribution of proteins within manually annotated functional classes. Proteins were categorized as invariant proteins (INV; white) or proteins with higher abundance or exclusive presence in the mature green (MG30; green) or red ripe (R45; red) stage of fruit development.

**Figure 4:**
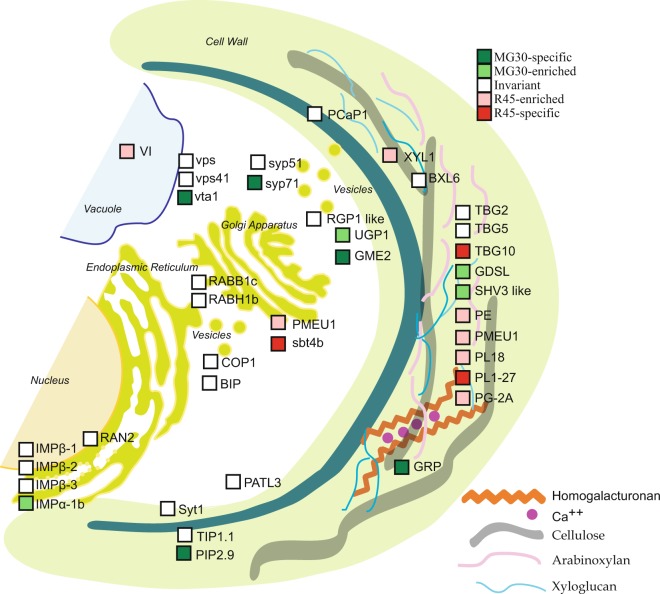
Schematic view of the proteins involved in cell wall metabolism, transport and vesicular trafficking. The colored boxes indicate differential or invariant proteins based on proteomic data. Proteins are shown in their final localization. GME2, UDP-glucuronate 4-epimerase 4; GRP, Glycine-rich protein; UGP1, UTP-glucose 1 phosphate uridylyltransferase: GDSL, SHV3 like, GDSL esterases; PE, PMEU1, Pectinesterases; PL18, PL1–27, Pectate lyases; TBG5, TBG10, Beta-galactosidases; BXL6, beta-D-xylosidase; XYL1, alpha-xylosidase 1; RGP1 like, Beta-1 4-glucan protein synthase; PCaP1, plasma membrane-associated cation-binding protein 1; IMPα-1b, IMPβ-2, IMPβ-3, IMPβ-1, Importins; RAN2, GTP-binding nuclear protein; RABB1c, RABH1b, Rab GTPases; RGP1 like, Beta-1 4-glucan protein synthase; COP1 (B-COP-Iα1, B-COP-Iα2, F-COP-Iβ, F-COP-IΥ), coat protein complex-I subunits; BIP, Luminal-binding protein; Syt1, Syt1 synaptotagmin; syp51, syp71, Syntaxins; PIP2.9, TIP1.1, aquaporins; PATL3, Patellin-3; sbt4b, Subtilisin-like protease; VI, vacuolar invertase; vps, vps42, vta1, Vacuolar protein sorting-associated proteins.

**Figure 5:**
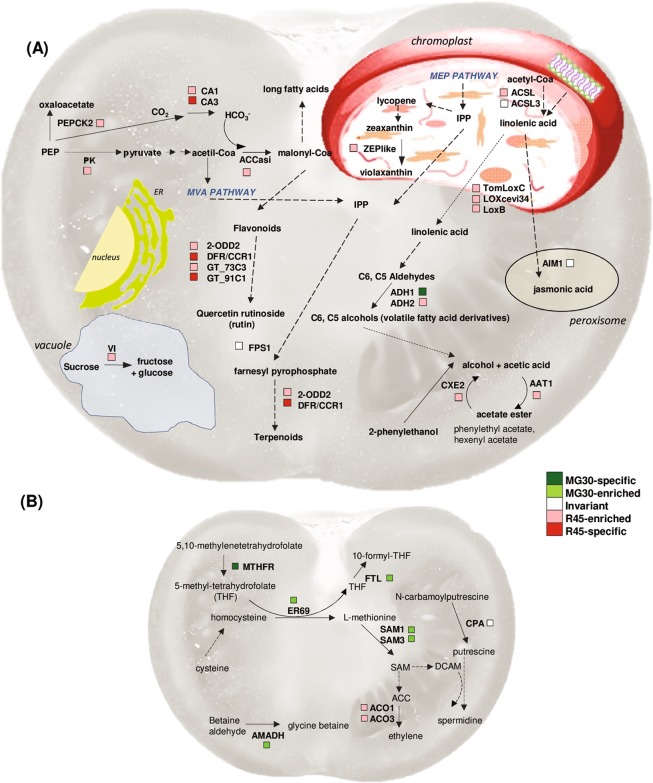
Changes in secondary metabolism, lipid metabolism and hormone biosynthesis during tomato fruit ripening. (**A**) Lipid metabolism and production of aroma compounds. (**B**) The increase in the SAM pool and ethylene production during tomato fruit ripening. The colors indicate the differential or invariant proteins based on proteomic data. AAT1, Alcohol acetyltransferase; ACCasi, Acetyl-CoA carboxylase; ACSL, ACSL3, Long-chain-fatty-acid–CoA ligase; ACO1, ACO3, 1-aminocyclopropane-1-carboxylate oxidase; ADH1, ADH2, Alcohol dehydrogenase; AIM1, Fatty acid oxidation complex subunit alpha; AMADH, Betaine aldehyde dehydrogenase; CA1, CA3, Carbonic anhydrase; CPA, N-carbamoylputrescine amidase; DCAM, Decarboxylated-SAM; DFR/CCR1, Cinnamoyl CoA reductase-like 1; CXE2, Acetyl esterase; ER69, homocysteine methyltransferase; FTL, Formate-tetrahydrofolate ligase; FPS1, Farnesyl pyrophosphate synthase; GT_73C3, GT_91C1, UDP-glucosyltransferase LOXcevi34, LoxB, LoxC, Lipoxygenase; 2-ODD2, 2-oxoglutarate-dependent dioxygenase; MTHFR, Methylenetetrahydrofolate reductase; PEPCK2, phosphoenolpyruvate carboxykinase; PK, pyruvate kinase; SAM1, SAM3, S-adenosylmethionine synthase; VI, vacuolar invertase, ZEPlike, zeaxanthin epoxidase like; PEP, phosphoenolpyruvate.

**Figure 6:**
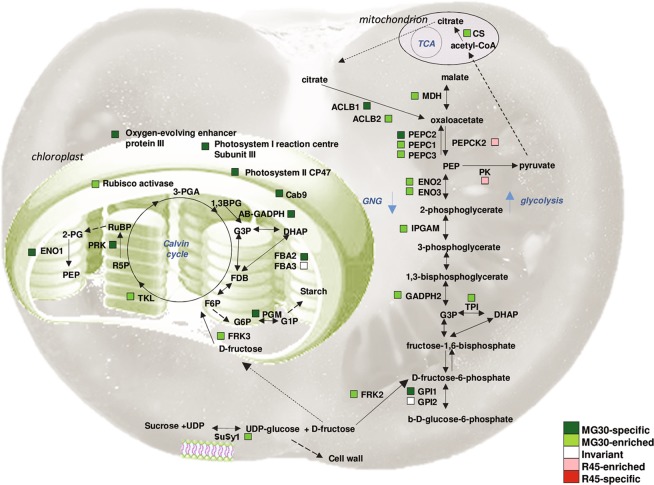
Metabolic pathway scheme summarizing changes in carbon, energy and photosynthesis metabolic pathways during tomato fruit ripening. The colors indicate differential or invariant proteins based on the proteomic data. The broken arrows indicate multiple steps between the two compounds. AB-GADPH, Glyceraldehyde 3-phosphate dehydrogenase; GADPH2, NADP-dependent glyceraldehyde-3-phosphate dehydrogenase isoform; ACLB1, ACLB2, ATP citrate lyase a-subunit; CS, Citrate synthase; ENO1, ENO2, ENO3, Enolase; FBA2, FBA3, Fructose-bisphosphate aldolase; FRK2, FRK3, Fructokinase; GPI1, GPI2, Glucose-6-phosphate isomerase; MDH, malate dehydrogenase; PEPC1, PEPC2, PEPC3 phosphoenolpyruvate carboxylase; PEPCK2, phosphoenolpyruvate carboxykinase; PGM, Phosphoglucomutase; PK, pyruvate kinase; TPI, Triosephosphate isomerase; Susy1, Sucrose synthase; TKL, Transketolase 1, 1,3BPG, 1,3-Bisphosphoglycerate; RuBP, Ribulose-1,5-bisphosphate; R5P, ribulose-5-phosphate; FDB Fructose-1,6-Bisphosphate; F6P fructose-1,6-bisphosphate; 2PG, 2-phosphoglycerate; PEP, phosphoenolpyruvate, DHAP, dihydroxyacetone phosphate.

Biological processes that were over-represented among proteins with increased abundance in MG30 fruits were:,monosaccharide metabolic process, cellular amino acid biosynthetic process, cellular amino acid metabolic process, carbohydrate metabolic process, glycolysis, and generation of precursor metabolites and energy. Proteins with increased abundance in R45 fruits were enriched in lipid metabolic process (Fig. 3A). The quantified proteins were grouped into fourteen broad functional classes that were manually compiled by grouping GO terms relative to different biological processes (Fig. 3B and Table S3). Presence of the identified proteins in known biological pathways was analyzed using the metabolic maps provided by the SolCyc database. Proteins associated with cell wall metabolism, transport and vesicular trafficking are illustrated in Fig. 4. Other proteins involved in lipid metabolism, secondary metabolism and in ethylene production are visualized in Fig. 5; proteins involved in C compound, energy and photosynthesis metabolic processes are illustrated in Fig. 6.

### Correlation analysis of protein and transcript abundance

**Figure 7:**
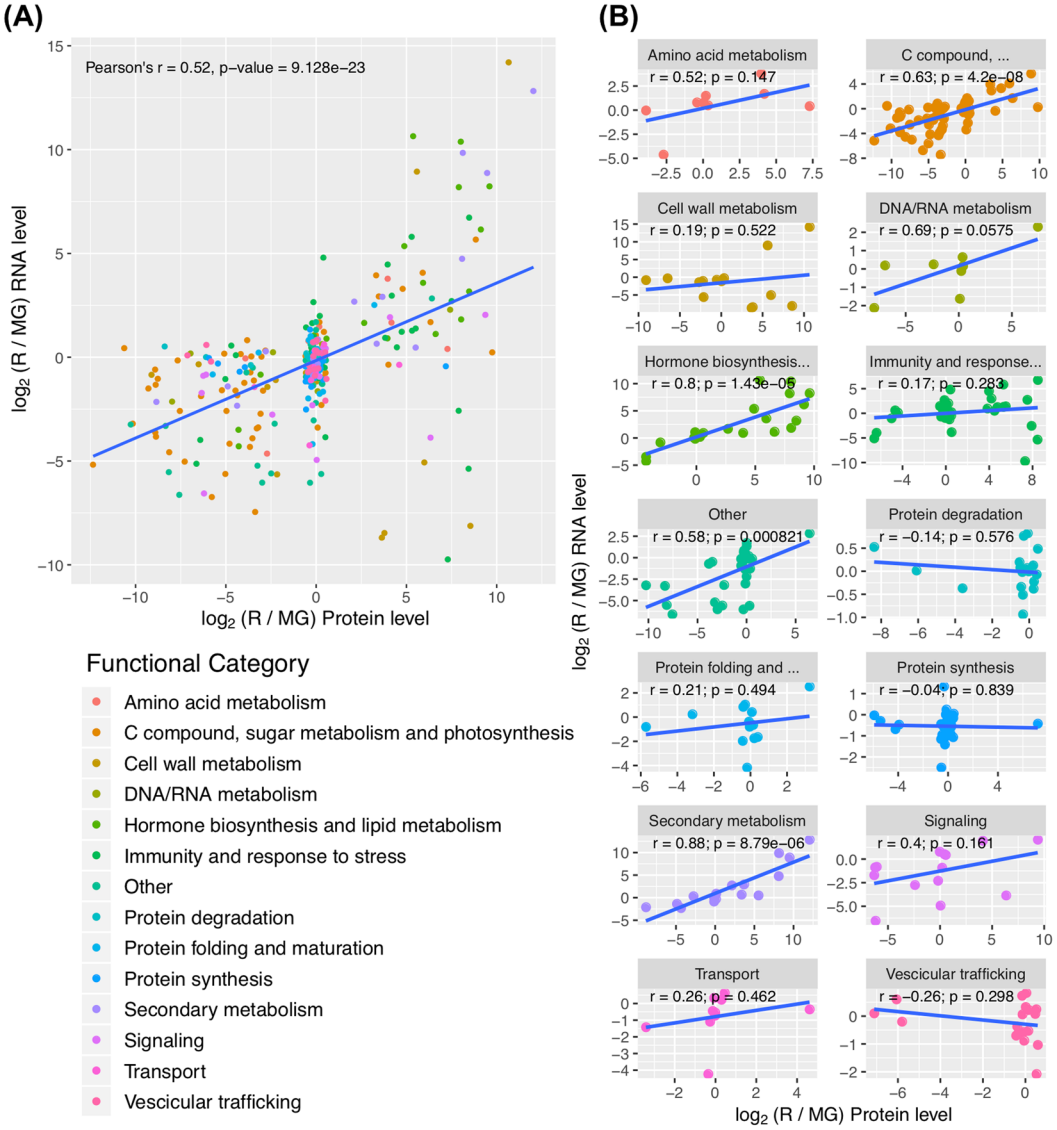
Correlation of differences in mRNA and protein levels for the quantified proteins. Values represent the log2 ratio of mRNA and proteins for R45 vs MG30 fruits (listed in Supplementary Table S3). (**A**) Correlation plot for genes classified in functional classes. The r values are calculated separately for each functional class. (**B**) Global correlation plot for all the 304 quantified proteins.

The possible correlation of protein abundance with mRNA abundance obtained from available transcriptomic datasets was analyzed both globally and for the fourteen manually compiled protein classes (Fig. 7 and Table S3). Transcript levels data of red ripe and mature green stages were from the RNA sequencing work of the Tomato Expression Atlas (TEA) database^24^.

The global analysis showed a relatively low global Pearson correlation coefficient (0.52) (Fig. 7A, with the slope of the linear regression line in the log/log-scaled scatter plot reflecting the differences in dynamic range between transcript and protein changes. Analysis of the fourteen classes (Fig. 7B) revealed high heterogeneity among them, with some classes showing higher correlation values [Hormone biosynthesis and lipid metabolism (r = 0.8); Secondary metabolism (r = 0.88), C compound, sugar metabolism and photosynthesis (r = 0.63)] and others showing virtually no correlation [Protein synthesis (r = −0.04)]. Correlations were significant (P < 0.05) for two categories that comprised a high number of genes [C compound, sugar metabolism and photosynthesis, (62 genes); Other (30 genes)], but also for two categories with a lower number of genes [Hormone biosynthesis and lipid metabolism (21 genes); Secondary metabolism (16 genes)]. All the other categories, with number of genes ranging from 8 to 40, showed no significant correlations (P > 0.05).

## Discussion

Changes of microsomal proteins and how they relate to the metabolic shift during fruit ripening have been here investigated. GO term enrichment analysis suggests that our set of identified proteins are enriched in organelle proteins and proteins located in the endomembrane system (plasma membrane, vacuole, membrane, cell wall, endosome, endoplasmic reticulum, whole membrane, Golgi apparatus part). Cytosolic and ribosomal proteins were also enriched. In agreement with previous reports^25^, the enrichment in ribosomal proteins is not unexpected, due to the interaction of ribosomes with the rough RE. On the other hand, the preparation of microsomal fractions for proteomic analysis is known to be challenging due to the difficulty of extracting low abundance proteins and at the same time maintaining membrane integrity. A large proportion of cytosolic proteins have been identified in multiple subcellular compartments, including endomembranes, especially in proteomics analyses^26^. True interactions with membranes or membrane proteins during folding processes may explain enrichment of cytosolic (and ribosomal) proteins. Importantly, a large number of proteins that are predicted to be cytosolic are recruited in macromolecular complexes associated to membranes, including those of the ER^27^, to form those microcompartments that are called “metabolons”^28,29^. These metabolic complexes offer the advantage of improving reaction specificity and flux routes through substrate channelling. For example, there is strong evidence for the participation in metabolons of the operationally soluble enzymes of the flavonoid branch pathway^27,30,31^ and glycolysis^32^.

Indeed, glycolysis was among the biological processes that were over-represented in our set of proteins, along with monosaccharide metabolism, cellular amino acid biosynthesis, cellular amino acid metabolic process, carbohydrate metabolism and generation of precursor metabolites and energy, in MG30 fruit. Lipid metabolism was instead enriched in R45 fruits (Fig. 3A). These processes, discussed in more detail below, are related to the main changes associated with ripening that affect taste (increase in sugar and decrease in organic acids), flavour (production of volatile compounds providing the characteristic aroma), firmness (softening by cell wall-degrading enzymes).

### Correlation between transcriptomic and proteomic changes

Studies comparing the overlap in differential expression patterns from both transcript and protein profiling in Arabidopsis have reported different congruence values, defined as the fraction of genes that exhibit a significant correlation (P < 0.05). Congruence values ranged from a very low fraction^33^ to more than one half^34,35^. Correlation appears to be dependent on the specific growth and development conditions^34^ and highly variable in different ontologic gene categories^35^, besides being influenced by technical problems related to the efficiency of purification in case of proteins localized in specific cellular compartments (i.e. membrane) at different growth stages.

Although, globally, in this work we found a poor correlation between transcriptomic and proteomic changes during tomato maturation (Fig. 7), the integration of transcriptomic and sub-cellular proteomic data for the different biological processes revealed different trends. Some ontological categories (secondary metabolism, hormone biosynthesis and lipid metabolism, C compound, sugar metabolism and photosynthesis) showed a good correlation (Fig. 7B). In general, the ontological categories subjected to fast dynamic regulation are more prone to mRNA/protein abundance correlation, as suggested by previous studies in other organisms such as *Saccharomyces cerevisiae*^36^ and *Drosophila melanogaster*^37^. In the latter case, mRNA and protein levels showed a fairly good Pearson correlation coefficient of r = 0.8, when genes not responding to the applied stimulus were removed from the analysis.

Categories such as vesicular trafficking, transport, protein synthesis and protein folding and maturation instead showed, in most cases, a relatively invariant protein abundance accompanied by a transcript increase that, however, was not higher than 2-fold (Fig. 7B). In these cases, transcript regulation may be unsuitable, albeit unavoidable, because of the broad action of transacting factors, whereas protein abundance remains stable, being likely controlled by translational and post-translational regulatory mechanisms, as previously suggested^38^.

Discrepancy between protein abundance and mRNA abundance may indicate mechanisms that differentially control protein abundance such as protein degradation or changes in protein localization, the latter meaning that the increased abundance of a protein in the membrane compartment might be counterbalanced by a decrease in another compartment. Therefore some changes observed here in microsomal fractions may not be detectable in total extracts. On the other hand, decreased transcript levels corresponding to invariant protein abundance can be explained by high protein stability. Post-translational modification (PTM) and selective degradation may also explain constant transcript levels accompanied by decreased protein levels.

### Cell wall metabolism

Proteins enriched in the MG30 fruit included proteins involved in synthesis and remodeling of the cell wall such as GME2 (UDP-glucuronate 4-epimerase 4), which plays a role in the dimerization of pectin rhamnogalacturonan-II^39^, UGP1 (UTP-glucose 1 phosphate uridylyltransferase), involved in the biosynthesis of glucans^40^, and two esterases [GDSL (Solyc01g098650) and SHV3-like (Solyc12g014570)]. A rice GDSL homolog deacetylates the hemicellulose xylan in rice^41^, while one in Arabidopsis (SHV3) is involved in pectic polysaccharide cross-linking^42^. A glycine-rich protein (GRP) that may participate in cell wall cross-linking or act as pectin plasticizer^43^ was also more abundant in MG30 fruit (Fig. 4).

During ripening, coordinated events of modifications of the primary cell wall and middle lamella polysaccharides result in softening and swelling of the wall, which, combined with alterations in turgor, bring about fruit softening and textural changes. The earliest events are usually a loss of pectic galactan side chains and the depolymerization of matrix glycans, which may begin before ripening, followed by a loss of pectic arabinan side chains and pectin solubilisation. Pectin depolymerization begins during early to mid-ripening, but is usually most pronounced late in ripening^44^. Indeed, in R45 fruits we found increased abundance of several apoplastic proteins involved in pectin degradation and modification: two pectate lyases (PL18, PL1–27), the polygalacturonase PG-2A and a beta-galactosidase (TBG10), all already associated with changes in fruit texture and taste during ripening, and two pectinesterases [PE, previously detected in the secretome of tomato fruits only at the red ripe stage^45–48^, and the pectin methylesterase (PME) PMEU1). Two invariant beta-galactosidase isoforms (TBG5 and TBG2) have been also quantified in our study. Beta-galactosidases hydrolize the galactosyl residues mainly from the pectin of the cell wall, leading to the accumulation of free galactose. Interestingly, galactose, when injected into tomato fruits, induces ethylene production and promotes early ripening, thus acting as a regulatory molecule^49^. Attempts to reduce polygalacturonase activity had relatively little effect on fruit softening^50^, while more significant effects were obtained by silencing PL18^51,52^ or beta-galactosidase^53^ expression.

The removal of methyl groups from pectin by pectinesterases, also found more abundant in the R45 fruit, is important during ripening: PME activity is higher in red ripe fruit compared to mature green and breaker stage, and a corresponding decrease in the degree of pectin methylesterification is observed^54^. The demethylesterified homogalacturonan can either form Ca^2+^ bonds, which promote the formation of the so called “egg-box” gel structures, or become a target for polygalacturonases and pectate lyases, affecting the texture and rigidity of the cell wall^55^. It has to be noted that a remarkable inverse correlation exists between transcript and protein levels for PL1–27, PE, PMEU1 and the TBG10.

We also found a α-xylosidase (Xy11), which removes xylosyl residues from xyloglucan (a hemicellulose). Interestingly, the Arabidopsis homolog of Xy11 was identified in a proteomic study of apoplastic protein changes in response to oligogalacturonides, pectin fragments released by the action of polygalacturonases^56^ that are capable of eliciting plant defense responses and antagonizing auxin responses^57,58^. Oligogalacturonides also lead to ethylene synthesis in tomato fruit, by up-regulating the genes encoding 1-aminocyclopropane-1-carboxylate (ACC) synthase 2 (*ACS2*) and the ACC oxidase 1 (*ACO1*)^59^. These observations point to a very complex regulatory circuitry, involving also cell wall fragments that are generated during the ripening process.

The presence of cell wall modifying enzymes in our microsome preparations is expected, since they transit through the endomembrane system. Moreover, some of them are known to be retained in the Golgi or other compartments of the secretory pathway, as inactive pre-proteins, prior to the secretion of the processed, active mature polypeptides: this retention may prevent precocious attack of the cell wall polymers. PMEU1, which is more abundant in R45 fruit microsomes, belongs to the type-I PMEs, characterized by an N-terminal pro region that exhibits homology with pectin methylesterase inhibitors (PMEIs) and is thought to have an autoinhibitory function. The pro region is involved in subcellular targeting, by mediating the retention of unprocessed PME in the Golgi^60^, and is likely released from PME during maturation. The identified peptides showed the presence of the pro region in PMEU1 (Fig. S1); this may reflect the association of this enzyme to the endomembrane system, prior to the post-translational proteolytic processing that allows the release in the apoplast of the active enzyme at later developmental stages. A subtilase enzyme (SBT4b), specific for the R45 stage, may be involved in the maturation of PME by cleaving after the dibasic site RRLL within the proprotein. This enzyme shares 84% sequence identity with SBT3^61^, a subtilase that affects PME activity and pectin methylesterification levels in tomato plants^62^. The Arabidopsis homolog SBT3.5 can indeed process PME17, releasing the mature form into the apoplast^63^, indicating that these proteases play a role in the processing and activation of the pectic enzymes responsible for softening.

### Vesicular trafficking

Vesicle transport ensures the exchange of macromolecules and proteins between different cellular compartments and the endomembrane system^64^. Two proteins show increased abundance in the MG30 fruits: syntaxin SYP71, a plant-specific SNARE protein the role of which is still poorly characterized, and VTA1, a protein associated to vacuolar sorting proteins. The Arabidopsis homolog AtSYP71 possesses dual localization to the plasma membrane and the ER and may participate in yet uncharacterized traffic routes that may occur between the ER and PM^65^. A possible role for VTA1 may be related to vesicle trafficking involving vacuolar sorting and cargo proteins, important also for the accumulation of phenolic compounds, particularly flavonoids, in the vacuole^66^.

Invariant proteins include alpha, beta and gamma subunits of COP-I, the protein complex that coats vesicles transporting proteins from Golgi back to the ER, and between Golgi compartments^64^, and two Rab GTPases (RABB1c and RABH1b), a class of important determinants of membrane identity and membrane targeting^67^. The complex metabolic changes that occur during both maturation and ripening, for example in the cell wall, requires also a tight control of the trafficking routes and it is not surprising that the levels of these proteins are maintained constant throughout the ripening process. Perturbation of the trafficking process has a great effect on the fruit phenotype: silencing of the Rab11 GTPase gene leads to reduced levels of pectinesterase and polygalacturonase in the fruit and delayed softening^68^, as well as an increased proportion of cellulose concomitant to a reduction in pectin^44^. SlRab11a may therefore be important for pectin trafficking during fruit development.

### Lipid metabolism and production of aroma compounds

The aroma is one of the most important quality attributes of tomato, since volatile compounds can be sensory indicators for nutritional and health value^69^. Over 400 aroma volatiles have been identified in tomato fruit^70,71^, but only a limited number of them, such as the C6-compounds hexanal, trans-2-hexanal, hexenol, cis-3-hexanol, and the C5-compounds 3-methylbutanal, 3-methylbutanol and 3-methylnitrobutane are the principal contributors to flavor^72^, whereas volatile ester are present at relatively low levels^73^. These characteristic tomato aromas are formed by several different processes, i.e. the lipoxygenase (LOX) pathway, the main process responsible for the production of aromas^74,75^, deamination and decarboxylation of amino acids, and oxidative cleavage of carotenoids^74^.

LOX catalyzes the dioxygenation of polyunsaturated fatty acids (e.g. linoleic acid and linolenic acids)^76,77^, leading to generation of two possible products, the 9- and 13-hydroperoxide, which serve as substrates for hydroperoxide lyase (HPL) to produce aldehydes (hexanal and cis-3-hexenal) that are further converted by alcohol dehydrogenase (ADH) into hexenol, cis-3-hexenol and others^71^. In our proteomic analysis, we detected up-regulation at both the protein and transcript levels of three lipoxygenases [LOXcevi34 (Solyc01g099160); LoxB (Solyc01g099190); TomLoxC (Solyc01g006540)] of the tomato LOX family. The 13-LOX TomLoxC is predicted to be chloroplastic and essential for generation of fruit C5 such as 1-penten-3-ol, 1-penten-3-one, pentanal, (Z)-2-penten-1-ol, and 1-pentanol)^78^ and C6 flavour volatiles^79^ (Fig. 5A).

Two ADHs have been identified in tomato: ADH1, found only in pollen, seeds and young seedlings, and ADH2, shown to accumulate during the later stages in ripening concomitant with the accumulation of flavor volatiles^70^. We found both, ADH1 as present only in MG30 fruits, and ADH2 as more abundant in R45 fruit, confirming the role of these enzymes in fruit development. Overexpression of ADH2 in fruit leads to an increase of the levels of C6 alcohols relative to C6 aldehydes, and of a ‘ripe fruit’ flavor, attributed specifically to increased levels of Z-3-hexenol^80–82^. The levels of volatiles in fruits are also regulated by the action of AAT1 (alcohol acetyltransferase) and CXE2 (acetyl esterase), both enriched in the R45 stage^73,83^.

### Amino acid metabolism

Two glutamate decarboxylases showed an opposite behaviour in their abundance (GAD2, MG30-enriched; GAD1, R45–enriched) that well correlated with their mRNA levels, suggesting a fine control of their product, the four-carbon non-proteinogenic amino acid gamma-aminobutyric acid (GABA). For this compound, a role in cellular pH regulation, promotion of glutamate transport from source organ to fruit and defense against pathogens has been proposed^84^. Its homeostasis is regulated during fruit ripening by the pathway known as GABA shunt, in which the GABA is converted to succinate and used as a substrate for respiration by the TCA cycle^85^. GABA levels are higher in mature green fruits, probably to protect immature seeds against pathogens, and rapidly decline during ripening when seeds have already matured. In parallel the levels of glutamate and/or aspartate [synthesized from glutamate through aspartate aminotransferase (Solyc08g041870), which was also identified among the putatively invariant proteins], dramatically increase.

Glutamate is also the precursor of proline, a compatible osmolyte that plays a protective and ROS scavenging role. A gamma-glutamyl phosphate reductase (Solyc06g019170), present in red fruit only, is also likely associated to proline biosynthesis. Glutamate is a precursor of proline also through the ornithine pathway^86^, and we found constant levels of two acetylornithine deacetylase isoforms (Solyc08g076990, Solyc08g076970), which catalyze the last step of ornithine formation, suggesting that this pathway is well active in tomato fruit. Ornithine is also a precursor of the polyamine putrescin, from which the other two common polyamines, spermidine and spermine, are formed. Polyamines are “compatible solutes” or “osmolytes” used to combat the environmental stresses^87^ and their role in tomato fruit ripening has been recently reviewed^88^. Another well-known osmolyte compound is glycine betaine, which also plays a role as an osmoprotectant by stabilizing the structure of both proteins and membranes^89^. We found two enzymes implicated in the synthesis of these two classes of compounds: N-carbamoylputrescine amidase (CPA), invariant in the two stages analyzed, and betaine aldehyde dehydrogenase (AMADH), enriched in MG30 (Fig. 5B).

### Hormone biosynthesis

Tomato is a climacteric species in which ripening is associated to a burst in respiration and ethylene production. In this work we identified two S-adenosylmethionine synthase (SAM1 and SAM3) and two 1-aminocyclopropane-1-carboxylate oxidase (ACO1 and ACO3) involved in ethylene biosynthesis (Fig. 5B). Consistent with the general knowledge that ethylene production dramatically increases concurrently with the breaker stage^14^, SAM1 and SAM3 were more abundant in the MG30 stage. ACO1 and ACO3, the transcripts of which accumulate after the breaker stage^14^ instead showed higher levels in the R45 stage. These two enzymes have been reported to be localized at the plasma membrane^90,91^ besides the cytosol.

Other proteins more abundant in MG30 fruits and linked to the production of ethylene (Fig. 5B) are homocysteine methyltransferase (ER69, ethylene-responsive methionine synthase), methylenetetrahydrofolate reductase (MTHFR) and formate-tetrahydrofolate ligase (FTL), all involved in the biosynthesis of methionine. The gene *ER69* is preferentially expressed in fruit (mostly in mature green, breaker and turning) and is likely to encode a tomato cobalamine-independent methionine synthase that transfers the methyl group from N5-methyl-tetrahydrofolate to homocysteine^92,93^. Tetrahydrofolate (THF) and its derivatives, known as folates, are also required for the synthesis of ethylene and metabolites such as nicotinamide and polyamines^94^.

On the other hand, proteins enriched in the R45 stage include the 2-ODD (2-oxoglutarate-dependent dioxygenase) that participates in the synthesis of ethylene, gibberellic acid, brassinosteroids^11^, and flavonoids^95,96^, and a chloroplastic zeaxanthin epoxidase, ZEPlike, that converts the carotenoid zeaxanthin into violaxanthin, precursor for the synthesis of the hormone ABA^97^. Indeed, a peak of ABA occurs during softening^98^. As an invariant protein we identified AIM1 **(**abnormal inflorescence meristem), the peroxisomal-located enzyme whose homolog in *A. thaliana* is implicated in a β-oxidation reaction in jasmonic scid biosynthesis^99^ (Fig. 5A).

### Protein synthesis, Protein folding and maturation, Protein degradation

The majority of the proteins that fall within these classes are invariant, indicating that these processes are constitutive throughout ripening. The few differential proteins show higher abundance in the MG30 fruits, except for the plastid ATP-dependent chaperone ClpB, the level of which increases in the red fruit. ClpB expression is induced by several stresses (e.g. heat shock in tomato), and damaged proteins may undergo subsequent refolding through ATP-dependent chaperones such as the DnaK system or ClpB/DnaK^100^.

### Interconversion of sugars and acids

The balance between sugars and acidic compounds greatly influences the development and maturation as well as the flavour of tomato fruit. In our dataset of differential proteins, several enzymes that take part in sugars and acid interconversion pathways [e.g. glycolysis, tricarboxylic acid cycle (TCA) cycle and gluconeogenesis (GNG)] are found (Table S2 and Fig. 6).

Glycolysis and GNG share many enzymes and are reciprocally regulated during tomato fruit ripening; in the MG30 stage, several shared enzymes show much higher abundance: enolase (cytosolic isoforms ENO2 and ENO3), triose-phosphate isomerase (TPI) and the phosphoglycerate mutase (IPGAM). Three isoforms of PEPC, a cytosolic enzyme that uses bicarbonate to form oxaloacetate (OAA) through the irreversible β-carboxylation of the glycolysis intermediate phosphoenolpyruvate (PEP), and a cytosolic NAD-dependent malate dehydrogenase (MDH), which converts OAA to malate, also showed higher abundance. OAA can be converted to citrate in the mitochondrion to replenish the TCA cycle intermediates consumed during biosynthesis^101^, or used for amino acid biosynthesis. Malate and citrate are crucial for fruit acidity and can be stored in the vacuole in large amounts; they are also important to sustain the osmotic potential that allows rapid cell expansion in developing fruits^102^.

In the MG30 fruit, we found a higher abundance of subunits of the cytosolic ATP citrate lyase (ACLB1 and ACLB2), which is part of the citrate-malate-pyruvate shuttle system and forms acetyl-coenzyme A (acetyl-CoA) and OAA from citrate produced by the TCA cycle (Fig. 6). This conversion of a tricarboxylic acid into a dicarboxylic acid leads to a decrease in fruit acidity. Acetyl-CoA is also used for the synthesis of flavonoids, isoprenoids and malonate derivatives^72^.

During ripening, sugars, mainly glucose and fructose, accumulate in the pericarp, whereas organic acid content decreases^72^, although during the final stages citrate levels return high. R45 fruits showed increased abundance of phosphoenolpyruvate carboxykinase (PEPCK), which converts OAA to PEP + CO_2_ in an early and rate-limiting step in GNG, important for the accumulation of soluble sugars and dissimilation of organic acids^103^, with part of the malate used in gluconeogenesis and converted into sugar. Indeed, transgenic tomatoes with reduced levels of PEPCK^104^ show reduced levels of glucose and fructose during fruit ripening, and an accumulation of malate, providing evidence for gluconeogenesis from organic acids and a role of PEPCK in this process. A pyruvate kinase (PK), which convert PEP to pyruvate in the last step of glycolysis (Fig. 6) increased in the mature fruit, likely related to the respiration increase that is typical of climacteric fruit.

The presence in our samples of many glycolytic, gluconeogenic and other enzymes predicted to be soluble may be explained by their organization in “metabolons”^28,29^ for channeling of metabolic intermediates.

Two cytosolic isoforms of carbonic anhydrase (CA1 and CA3) were also detected (Fig. 5A). CA1 was strongly enriched in R45 fruits while CA3 appeared to be specifically present in R45 fruits, likely related to the high respiration rate. As the tomato fruit has a relatively thick cuticle and no stomata, gas exchange should be extremely low, and CO_2_ accumulating inside the fruit could be reused in the fruit, for example by CAs, which convert it to bicarbonate, substrate for PEPC. Only for CA1 there was a good correlation with the transcript levels. In has to be noted that in a previous work, transcripts of *CA1*, but not of *CA2* and *CA3*, were detected in mature green fruits^105^.

### Sucrose metabolism

Sucrose is translocated to the fruit, a sink organ, and degraded. As a sucrose turnover enzyme, sucrose synthase 1 (SuSy1), a glycosyltransferase that converts sucrose into UDP-glucose and fructose in the presence of UDP, was found enriched in MG30 fruit, with a good correlation with transcript levels (Fig. 6). A key function of SuSy is its contribution to cell wall formation by providing UDP-glucose for the synthesis of cellulose, necessary during fruit development, and callose^106^. SuSy can be associated to plasma membrane, as well as to vacuoles and mitochondria^107^.

A fructokinases (FRK2) that may convert fructose formed by SuSy to F6P is also more abundant in MG30 fruits, together with GPI1, a phosphoglucose isomerase, which may convert F6P to G6P. The co-expression of *FRK2* and *SUSY* genes in green fruits has been proposed to be necessary to maintain SuSy activity, which is inhibited by free fructose^108^. FRK2 is not required for starch synthesis in young tomato fruits, since *FRK2* silenced plants displayed even slightly higher levels of starch^109^, rather appears important for cell wall synthesis^110^. Also FRK3, the only plastid FRK found in tomato, is increased in MG30 fruits. However, a role in fruit development has not yet been ascribed to either FRK2 or FRK3, whereas their importance for cell wall synthesis during vascular development has been shown^110,111^. The phenotype of *FRK3* and *FRK3*x*FRK2* silenced plants indicate an additive effect of FRK3 and FRK2 suppression, suggesting that the plastid and cytosolic FRKs may partially compensate for each other, although they must have also unique functions^110^. The presence and source of fructose within plastids is not clear yet^110^. Sugars have also important functions as primary messengers in signal transduction and the phosphorylation by FRK could be important also in hexose sensing^112^.

Finally, the chloroplastic phosphoglucomutase (PGM)^113,114^, that is involved in starch biosynthesys, was present exclusively in MG30 fruits, consistently with the rapid decline in starch content that occurs during fruit ripening.

A sucrose turnover enzyme, a vacuolar invertase (VI), was enriched in R45 fruit, in accordance with the transcript level and with the notion that its gene is a direct target of the ripening regulator RIN (Fig. 5A)^115^. High VI activity in red fruit is necessary to maintain the cellular hexose concentrations, as its antisense suppression resulted in reduced hexose accumulation during fruit ripening^116^. The invertase activity is also controlled post-translationally by inhibitor proteins^115,117^. The decrease of SuSy and increase of VI during ripening may be important to regulate the balance between hexoses, as only VI produces free glucose and thus forms twice as many hexoses compared to the degradative action of SuSy. In addition, the vacuolar site of cleavage by VI could allow temporal control of hexose concentrations via compartmentalization^81,118^.

### Secondary metabolism

We found, enriched in the R45 fruit, a putative cytosolic acetyl-CoA carboxylase (ACC) and the CA1 and CA3 isoforms of carbonic anhydrase. ACC is an enzyme that uses bicarbonate to produce malonyl-CoA, which is used in both flavonoid biosynthesis and fatty acid elongation (Fig. 5A). Also CA1 and CA3 may be important for malonyl-CoA synthesis, because bicarbonate is used by not only by ACCs but also for the synthesis of oxaloacetate, which in turn is converted to malate, a key substrate for respiration during fruit ripening.

Soluble enzymes of the flavonoid branch pathway were identified in our study: chalcone isomerase (CHI, Solyc07g062030) and dihydroflavonol 4-reductase/cinnamoyl-CoA reductase (DFR/CCR1 Solyc04g082780). Flavonoid compounds are produced by the coordinate action of soluble and membrane-bound cytoplasmic enzymes. Physical interactions between the soluble enzymes of the flavonoid branch pathway was demonstrated using yeast-two-hybrid, immunological and physicochemical methods^27,119^, and these proteins have been proposed to be components of metabolons, probably by interacting with membrane-bound enzymes of the cytochromes P450 family^120^. In particular DFR/CCR1, detected only in the R45 stage, is a key enzyme in the flavonoid biosynthetic pathway that catalyzes the reduction of dihydromyricetin to leucodelphinidin, a precursor of anthocyanins that may act as frugivore attractants in ripe fruits, (Fig. 5A). More abundant or detected only in the R45 stage there were also two glycosyltransferases (GT_73C3 and GT_91C1) that might be involved in O-glucosylation of quercetin to yield the flavonol glycoside rutin (quercetin 3-rutinoside), which is the main flavonoid detected in the fruit flesh and shows increased levels during ripening.

### Immunity and response to stress and signaling

In the green fruit, the need for defense against ROS production associated to photosynthesis has been widely described and involves several enzymes such as catalase (CAT), superoxide dismutase (SOD), glutathione reductase, monodehydroascorbate reductase (MDAR) and dehydroascorbate reductase (DHAR). Conversion of chloroplast to chromoplast leads to a substantial change of the antioxidant apparatus, with a reduction in the activities of SOD, CAT, and most of the enzymes associated with the ascorbate–glutathione cycle^121^. Our results showing a decrease in red fruit of a DHAR (Solyc05g054760) and a SOD (SOD6) are in agreement with the reduction in ROS scavenging activity.

Protective enzymes against oxidative stress are also glutathione-S-transferases, which convert glutathione (GSH) to R-S-glutathione, and methionine sulfoxide [Met(O)] reductase. The latter, indicated also as ethylene-responsive gene E4^122^, was observed only in R45 fruits and acts in the reduction of Met(O) in proteins to methionine, likely to protect protein structures from oxidative damage^123^.

A divergent behavior was observed for two GST isoforms: Solyc07g056420, previously indicated as *LeGSTU2*^124^, is more abundant in the R45 fruit, whereas Solyc06g009020 prevails in the MG30, although its transcripts do not show a large variation between the two stages. The protection potential of *LeGSTU2* has been demonstrated in transgenic Arabidopsis plants, which exhibited enhanced resistance to salt and drought stress^124^.

Several defense proteins were observed in the red fruit: a defensin (Solyc07g007760, R45-specific), a homolog of the pathogenesis-related PR4 (Solyc01g097240, R45-enriched) and a metallocarboxypeptidase inhibitor (2A11, Solyc07g049140). PR4, similar to the antifungal chitin-binding protein hevein from rubber tree latex, has been shown to inhibit the growth of some pathogenic fungi; indeed, a purified PR4b protein from pepper inhibits spore germination and mycelial growth of plant fungal pathogens and its transient expression triggers hypersensitive cell death^125^.

Our results also indicate a change in the battery of proteases during tomato fruit formation and maturation that may be involved in protection against pests^126^. We find two putative cathepsin B enriched in the red fruit (Solyc02g069100, Solyc02g076710). Both may be involved in stress responses; for example, in Arabidopsis, cathepsin B have been shown to be involved in disease resistance^127^. A serine carboxypeptidase Solyc04g077650 also appears as R45 fruit-specific. Interestingly, the putative Arabidopsis ortholog AT3G12203/SCPL17 is an acyltransferase responsible for the modification of glucosinolates (GL), secondary metabolites that protect plants from pests^128^.

A number of differential proteins involved in signaling processes here identified have still no defined function and may be involved in stress responses. Among these, there are several receptor-like kinases [Solyc03g121050, a putative orthologue of Arabidopsis LYK3, involved in immunity^129^; Solyc03g118510, Solyc12g007110, MG30-specific; Solyc03g111670, MG30-enriched; Solyc11g056680, R45-enriched] and two transduction elements [Solyc12g057110, a cytoplasmic MG30-specific 14-3-3 protein; Solyc02g089150, a R45-specific protein containing a phosphoinositide phospholipase C X (PI-PLC X) domain].

In conclusion, our results complement previous extensive proteomic studies on tomato fruit, adding information on membrane proteome composition and changes during ripening. The extensive knowledge of metabolic pathways and protein complexes can be important prerequisites to further improvement of tomato fruits.

## Methods

### Growth conditions and experimental set up

Tomato plants (*Solanum lycopersicum* cv Money Maker) were cultivated using standard greenhouse conditions (26 °C and 12 h supplemental lighting, followed by 12 h at 20 °C). Tomato fruits were collected at 30 and 45 days post-anthesis (DPA), corresponding to the mature green and red tomato ripening stages (MG30 and R45), respectively. To maximize developmental synchrony, harvested fruit were visually inspected externally and internally for several features (e.g. size, shape, pigmentation, seed development, and development of locular jelly), and only fruit pericarp (the vast majority) appearing developmentally equivalent were considered for analysis. Experiments were performed in three biological replicates, with each replicate consisting of a pool of at least 5 fruits.

### Total membranes extraction and fractionation

Tomato pericarp was homogenized in homogenization buffer (0.25 M sucrose, 10 mM HEPES NaOH, pH 7.4, 1 mM EDTA, 1 mM DTT) at 4 °C in a tissue/buffer ratio of 1:1 (w/v), using a kitchen blender and three pulses of 10 seconds. The homogenate was centrifuged at 1000 × g for 20 minutes to remove cellular debris. Supernatant was collected, centrifuged at 10000 × g for 20 minutes to remove chloroplasts and the supernatant was centrifuged at 100000 × g for 2 h in a SW28 rotor. The pellet was recovered, resuspended in homogenization buffer and loaded onto a 6 ml 18% iodixanol cushion and centrifuged at 100000 × g for 2 h in a SW28 rotor (Beckman Coulter, Fullerton, CA). Total microsomes were then collected from the interface^130^ and stored at −80 °C. No additional washing steps were performed in order to minimize the disruption of membrane-associated supramolecular complexes (for example, metabolons).

### Western blot analysis

The fractions obtained from the extraction and fractionation procedure (total homogenate, supernatant of the second centrifugation, total microsomes recovered at the interface of the iodixanol cushion) were analyzed by Western blot with antibodies against different subcellular markers, essentially as described before^131^. Protein concentration was quantified by DC protein assay (BioRad, Richmond, CA) according to manufacturer’s instructions. Two μg of each sample were boiled in Laemmli buffer for 3 minutes, loaded on a 12% polyacrylamide gel and separated into an SDS-PAGE apparatus (Biorad). Subsequently blots were incubated with the different primary antibodies: anti-BiP (luminal binding protein, marker of the ER, kindly provided by Prof Alessandro Vitale, IBBA-CNR, Milan)^132^; anti-RGP (reversibly glycosylated protein, marker of the Golgi apparatus, kindly provided by Prof Alessandro Vitale, IBBA-CNR, Milan)^132^; anti-LHCII (light-harvesting complex II, chloroplast marker, kindly provided by Prof. Roberto Bassi, University of Verona); anti-AOX (alternative oxidase, marker of plant mitochondria, Agrisera antibody); anti-H3 (histone H3, marker of the nucleus, Thermo Fischer Scientific) at different dilutions depending on the antibody used (1: 10000 anti-BiP and anti-RGP1, 1: 500 anti-LHCII, anti-H3, anti-AOX) in washing buffer (1% PBS, 0.2% Tween20) containing 0.5% BSA, for 12 h at room temperature.

### Protein separation by 1D-SDS-PAGE and in gel digestion

An aliquot (30 µg total proteins) of each biological replicate in Laemmli buffer was heated at 37 °C to prevent aggregation of hydrophobic proteins, and then loaded on a 12% acrylamide gel for separation by 1D-SDS-PAGE. Proteins were visualized by Coomassie Brillant Blue staining. Each lane was divided into 10 gel slices of different mass range for in gel digestion performed as previously described^131^. Digests were acidified by addition of 1.5 µl of formic acid (5%, v/v) and desalted by C18 StageTips^131^ for LC-MS/MS protein identification. Digests from each gel slice were analysed in separate LC-MS/MS runs.

### Mass spectrometry analysis and protein identification

Mass spectrometry analysis was performed on a linear trap quadropole (LTQ) Orbitrap XL Discovery (Thermo Fisher Scientific) equipped with a nanoelectrospray ion source (Thermo) coupled to a Dionex Ultimate 3000 nanoflow LC system (Thermo Fisher Scientific). LC-MS/MS was performed essentially as described^131^, using a 160 minute multistep gradient of solvent A (5% ACN and 0.1% formic acid in milliQ-water) and B (80% ACN and 0.1% formic acid in milliQ-water).

### Protein identification

The raw data from the mass spectrometric analysis was processed using the MaxQuant software v. 1.3.0.5 supported by Andromeda^133^ as the database search engine for peptide identifications.

In the Quant module of MaxQuant, parameters were set as follows: protease specificity was set to trypsin and a maximum of 2 missed cleavages was allowed, filtering of MS/MS spectra to retain only the 6 most intense peaks per 100 Da, the first search peptide tolerance was set to 20 ppm, the main search peptide tolerance was set to 6 ppm and the MS/MS mass tolerance to 0.5 Da. Methionine oxidation and protein N-term acetylation were set as variable modifications and cysteine carbamidomethylation as fixed modification. The generated peak lists were searched against a forward and reversed version of the International Tomato Annotation Group (ITAG) 2.3 Tomato database containing 34727 protein entries, supplemented with known contaminants using the Andromeda search engine. Protein identification was performed in the MaxQuant Identify module using the following parameters: protein and peptide false discovery rate (FDR) < 0.01, posterior error probability based on Mascot score, minimum peptide length of 7, minimum peptides of 1, use of both razor and unique peptides for quantification. Additionally, we required at least two peptide identifications per protein, of which at least one peptide had to be unique to the protein group.

### Label-free quantification and statistical analysis

The three biological replicates for each developmental stage (five fruits for each replicate) were analyzed and subjected to label-free quantification (LFQ). The analysis was performed with MaxQuant and included quantification of peptides recognized on the basis of mass and retention time but identified in other LC−MS/MS runs. Feature-matching between raw files was enabled, using a retention time window of two minutes (“match between runs” option in MaxQuant). Data were evaluated and statistics calculated using the Perseus software (version 1.6.0.2, Max Planck Institute of Biochemistry, Martinsried). MaxQuant data were filtered for reverse identifications (false positives), contaminants and “only identified by site”. The LFQ intensities were log transformed.

For clustering analysis in the Perseus software, we considered proteins that had a LFQ intensity value different from 0 in at least 4 of the 6 total samples (3 biological replicates for each of the two stages), plus those that had valid values of LFQ intensity in all samples of the same developmental stage and a LFQ intensity value = 0 in all the samples of the other stage (3:0 or 0:3); these proteins are considered as stage-specific.

Log2 intensity values that were missing in 2 or 1 samples out of 6 were imputed using a downshifted normal distribution with width 0.3 and downshift 1.8 for each individual sample. Log2 intensity values that were missing in all three replicates of one condition (MG30 or R45 developmental stage), were imputed using the average minimum log-intensity across runs^134^.

Relative abundance ratios (the difference in average log2 intensity between R45 and MG30 samples) were calculated including the imputed values. Proteins were classified into five possible categories: MG-specific (proteins identified only in the MG30 sample), MG-enriched (log2 ratio ≤ −0.58, FDR < 0.05), invariant (−0.58 ≤ log2 ratio ≤ 0.58), R-enriched (log2 ratio ≥ 0.58, FDR < 0.05), and R-specific (proteins identified only in the R45 sample).

Those proteins that, in spite of a log2 ratio < −0.58 or > 0.58, did not pass the FDR < 0.05 threshold were classified as invariant.

The mass spectrometry proteomics data have been deposited to the ProteomeXchange Consortium via the jPOST partner repository [http://repository.jpostdb.org] with the dataset identifier JPST000400/PXD009132.

### Bioinformatics analysis of identified proteins

Protein descriptions were defined using annotations associated with each protein entry in the ITAG database (version 3.1). Homology-based comparisons with the TAIR10 protein database (http://www.arabidopsis.org) were performed using Basic Local Alignment Search Tool BLASTP^135^. For prediction of subcellular localization of transmembrane alpha helices, we used THMMM (http://www.cbs.dtu.dk/services/TMHMM/), which is based on hidden Markov models^136^, and CELLO v.2.5 (http://cello.life.nctu.edu.tw/). The prediction of an N-terminal signal peptide for translocation into the ER was done using the software SignalP [Predicting Secretory Proteins with SignalP (http://www.cbs.dtu.dk/services/SignalP/)]^137^.

Gene Ontology (GO) analysis was performed using Panther tools (PANTHER version 11: expanded annotation data from Gene Ontology and Reactome pathways, and data analysis tool enhancements^138^). Statistical over-representation analysis was performed to determine biological processes and cellular compartments in which the frequency of genes from our proteomic datasets was significantly greater than the frequency of genes in the *Solanum lycopersicum* reference database. The GO terms was considered statistically significant enriched when they had a corrected p-value < 0.05^139^.

The TOMATOMICS Database (http://bioinf.mind.meiji.ac.jp/tomatomics)^140^ was useful to find more information on annotated genes, and SolCyc (http://solcyc.solgenomics.net/overviewsWeb/celOv.shtml) was used to obtain detailed information on pathways and biochemical reactions involved.

For proteomic and transcriptomic data integration, RNA-seq data were obtained from the Tomato Expression Atlas **(**TEA) database (http://tea.sgn.cornell.edu)^24^. Normalized RPM values of Total_Pericarp:Red_Ripe (R45) and Total_Pericarp:Mature_Green_equatorial (MG30) from the database were used to obtain the relative fold changes. The mRNA-protein Pearson correlation coefficient (r) and relative p-values of log_2_ (R45/MG30) values were computed using *cor.test* function of R software (version 3.4.3) and *ggplot2* package (version 2.2.1) for scatter plots image. The mass spectrometry proteomics data have been deposited to the ProteomeXchange Consortium via the jPOST partner repository with the dataset identifier JPST000400/PXD009132^141^.

## Supplementary information

Figure S1

Table S1

Table S2

Table S3

DataSheet S1
