# Supplementary material for: Changes in the microsomal proteome of tomato fruit during ripening
Source: Sci Rep. 2019 Oct 4;9:14350. doi: 10.1038/s41598-019-50575-5 (PMC6778153; doi:10.1038/s41598-019-50575-5)
Supplement: Supplementary file 1 — Figure S1 [file 41598_2019_50575_MOESM1_ESM.pdf]

Supplementary Materials of “Changes in the microsomal proteome of tomato fruit during ripening”, by *Daniela Pontiggia, Francesco Spinelli, Claudia Fabbri, Valerio Licursi, Rodolfo Negri, Giulia De Lorenzo and Benedetta Mattei*

(A)

Accession	Coverage	description	N° peptides uniques
Solyc03g123630.2.1	34.5% (201/583)	Pmeu1	11

Sequence of identified unique peptides:

1. CDMLAYQDTLYVHSNR
2. DITFQNTAGASK
3. EHAEDLK
4. FIAGGSWLSSTGFPFSLGL
5. NVQDGSTTFHSATVAAVGEK
6. SNTIITASR
7. TDPNQNTGIVIQK
8. TLISSAITNQETCLDGFSHDEADKK
9. TNIMFMGDGK
10. VALHDCLETMDETLDELHTAVEDLELYPNKK
11. VITSSTEAQAYTPGR

(B)

*MTRVEDFFSKQIDFCKRKKKIYLAIVASVLLVAAV*IGVVAGVKSHSKNSDDHADIMAISSSAHAIVKS
ACSNTLHPELCYSAIVNVSDFSKKVTSQKDVIELSLNITVKAVRRNYYAVKELIKTRKGLTPREKVAL
HDCLETMDETLDELHTAVEDLELYPNKKSLKEHAEDLKTLISSAITNQETCLDGFSHDEADKKVRK
VLLKGQKHVEKMCSNALAMICNMTDTDIANEMKLSAPANNRKLVEDNGEWPEWLSAGD**RRLL**Q
SSTVTPDVVVAADGSGDYKTVSEAVAKAPEKSSKRYVIRIKAGVYRENVDVPKKKTNIMFMGDGK
SNTIITASRNVQDGSTTFHSATVAAVGEKFLARDITFQNTAGASKHQAVALRVGSDLSAFYRCDML
AYQDTLYVHSNRQFFVQCLVAGTVDFIFGNGAAVFQDCDIHARRPGSGQKNMVTAQGRTDPNQN
TGIVIQKCRIGATSDLRPVQKSFPTYLGRPWKEYSRTVIMQSSITDVIQPAGWHEWNGNFALDTLF
YGEYANTGAGAPTSGRVKWKGHKVITSSTEAQAYTPGR FIAGGSWLSSTGFPFSLGL

Figure S1. Sequence coverage of the pectimethylesterase PMEU1 identified in our study. (A) Total sequence coverage of PMEU1 (percentage of the protein sequence covered by identified peptides), N° unique peptides (number of peptide sequences that are unique to this protein) and sequence of identified unique peptides. **(B)** Identified peptides are highlighted on the amino acid sequence of PMEU1. Underlined sequence represents the putative N-terminal signal peptide. The sequences highlighted in light blue indicate peptides in the mature region, those highlighted in yellow indicate peptides in the N-terminal pro region and bold characters show the dibasic site RRLL recognized by proteases for cleavage of the pro-region.
